# Do species factories exist? Detecting exceptional patterns of evolution in the mammalian fossil record

**DOI:** 10.1098/rspb.2021.2294

**Published:** 2022-04-13

**Authors:** Jaakko Toivonen, Mikael Fortelius, Indrė Žliobaitė

**Affiliations:** ^1^ Department of Computer Science, University of Helsinki, Helsinki, Finland; ^2^ Department of Geosciences and Geography, University of Helsinki, Helsinki, Finland; ^3^ The Finnish Museum of Natural History, Helsinki, Finland

**Keywords:** macroevolution, net primary productivity, mammals, fossil record, NOW database, logistic regression

## Abstract

A species factory refers to the source that gives rise to an exceptionally large number of species. However, what is it exactly: a place, a time or a combination of places, times and environmental conditions, remains unclear. Here we search for species factories computationally, for which we develop statistical approaches to detect origination, extinction and sorting hotspots in space and time in the fossil record. Using data on European Late Cenozoic mammals, we analyse where, how and how often species factories occur, and how they potentially relate to the dynamics of environmental conditions. We find that in the Early Miocene origination hotspots tend to be located in areas with relatively low estimated net primary productivity. Our pilot study shows that species first occurring in origination hotspots tend to have a longer average longevity and a larger geographical range than other species, thus emphasizing the evolutionary importance of the species factories.

## 1. Introduction

While it is clear that rates of evolution have varied in the past [1], in which way they vary remains at the centre of palaeobiology research. Some places or times tend to give rise to more new species than others. To explain this Vermeij & Dietl [2] proposed the majority rule, which posits that the source of new or recovering populations is to be found primarily in productive and large environments. While not tested with data, intuitively, their hypothesis makes sense. Leaving aside complications due to dependence on carrying capacity [3] or diversity [4], environments that cover larger areas should accommodate more species [5] and thus, under fixed rates of evolution, could potentially produce more new species. The more new species are produced, the higher the chances are for them to spread and become common. How else could it be?

The concept of the species factory [6] describes quite the opposite phenomenon, where rather than originating from productive environments, many successful species originate from harsh or marginal environments [7,8]. Three examples of species factories in the mammalian record, the latest Middle Miocene origins of the Pikermian palaeobiome in the Sub-Parathetyan province [7], the Pliocene origin of Pleistocene megafauna in the Tibetan Plateau [9] and the rise of arid-adapted fauna in the Plio-Pleistocene of the Eastern African Turkana Basin [8], all point to trends of environmental change. The key condition is that marginal environments that are initially rare later become widespread, following persistent directional climate change, such as cooling or drying.

Here we ask whether some places, times, or combinations of places, times and types of environment give rise to abnormal numbers of new species, and if so, can we define and detect such hotspots in the mammalian fossil record computationally? We further ask whether species factories are just about luck, getting started ahead in environments that will later become widespread, or whether species factories could arise for other reasons. To answer this we search for exceptional patterns in the fossil record and analyse their contexts.

Even though the fossil record will remain incomplete [10,11], as more fossil data are accumulated and analysed, a more comprehensive picture emerges. In recent decades, fossil data from many sources have been put together to form large-scale fossil databases [12]. The availability of such vast resources has accelerated the development and usage of computational methods to analyse them, for instance, for tracking faunal communities and their dynamics [13–19], estimating biochronology of fossil localities [20–22], or reconstruction of their paleoenvironments [23–28].

Various techniques have been developed to make inferences on diversification rates that take the incomplete and biased sampling of the fossil record into account, such as sub-sampling [29,30] or capture–mark–recapture methods [31–36]. While most of the studies have concentrated on how origination and extinction rates have changed over time, spatial aspects are gaining attention as well, especially with availability of dedicated analysis toolboxes [37].

For analysing species factories our task is to detect anomalies in macroevolutionary patterns over space and time. We statistically detect exceptional origination, extinction and sorting patterns accounting for spatial resolution of the fossil record. By sorting we mean appearance of species that originated elsewhere and local extinctions. While many analyses of the mammalian record operate at the genus level [29,30,32,38], and there are good arguments for such choice [39], here we deliberately analyse the mammalian fossil record at the species level in order to reason about *species* factory phenomena. Our computational approach is thus tuned to work with species-level data that often lack precise taxonomic identification.

Our data analysis focuses on the Late Cenozoic record of mammalian species occurrences in Europe, reported in the NOW database of fossil mammals [40]. We flag origination, extinction and sorting hotspots in this region and analyse them in the context of palaeoclimatic conditions. For reconstructing palaeoclimatic conditions, we use existing dental ecometric models [27] that rely on composition of plant eating mammal communities and characteristics of their teeth.

In order to detect anomalies, we need baselines to quantify what patterns are normally expected, and we need to do it as locally as possible in time and space. For this purpose, we train a logistic regression model that gives us an expected number of occurrences of new species in a given place and time depending on sampling intensities in the current and the previous time unit. Then, we ask statistically, what is the probability to find this many or more occurrences of new species at any given locality in that time interval by chance. If that probability is low, we take that as a potential indicator of an exceptionally high origination rate at this place and time, and flag such a locality as a potential species factory. This approach extends naturally to flagging exceptional patterns of extinction and species sorting in space and time, which we analyse in the context of palaeoenvironmental reconstructions.

## 2. Study region and fossil data

We retrieved a public version of fossil data from the NOW database [40] on 26 April 2020. The data contained information on species occurrences at localities, stratigraphic context of localities, their age estimates, taxonomic affiliation of species and their ecological characteristics.

We delimited our analysis to Europe, similar to previous studies [41], as within −25° to 40° of longitude and latitude above 35°. However, we referred to the global data to determine globally first and non-first occurrences of species, as well as times of their global extinction.

We selected the localities falling within the age range of European Land Mammal ages (known as MN units) [42,43], covering age range from 23 Ma to 1.9 Ma [44] (MN1–MN17), we also included two informal MQ units that covered the time from 1.9 Ma to 0.01 Ma (MQ18–MQ19). All time bounds that we used are listed in the electronic supplementary material. We used MN and MQ units as time bins for the analysis. The analysis of species factories was on bins MN2–MQ18, while the first and the last bin was only used for determining the first and the last observed occurrences.

The selected snapshot of data contained 48 318 rows of data for the global dataset, that is 48 318 occurrences of 9692 unique species over 5360 localities. The spatially restricted dataset (only localities with longitude between −25° and 40° and latitude above 35°) included 22 939 occurrences of 2937 unique species over 2822 localities.

Data describing dental traits of the species came from the NOW database as well. We used the relative molar crown height (hypsodonty) and the number of longitudinal cutting edges (lophs) recorded at the species level. All the occurrences from species of the following orders were included in the dental-based environmental estimations: Perissodactyla, Artiodactyla, Primates, Proboscidea and Hyracoidea. Hypsodonty in NOW is reported as categories ‘bra’, ‘mes’, ‘hyp’ and ‘hys’, corresponding to brachydont, mesodont, hypsodont and hypselodont. We transformed this into an ordinal variable assigning values 1 and 2 for ‘bra’ and ‘mes’, respectively, and 3 for both ‘hyp’ and ‘hys’ following [25]. Longitudinal loph count in NOW ranged from 0 to 3 (many), but following [27], from which the predictive models come, we capped the number of longitudinal lophs at 2.

## 3. Computational methods

Our main methodological task is to define what makes the patterns of origination exceptional. The main challenge is varying sizes of the number of species at localities in time and space, which may be an artefact due to uneven sampling of fossil finds or it may be a genuine variation in diversity due to varying carrying capacity of the environment, or both. Rather than assuming and modelling sampling process for the purpose of data ‘correction’, we aim to directly model expectations for exceptional and non-exceptional patterns of occurrences along with any variations in sampling that there may be.

### (a) Detecting exceptional origination (species factories)

Our task is to detect areas in space and time that show exceptionally high amounts of first occurrences. We take this as a potential sign of origination hotspots (species factories).

All else being constant we would expect to see high numbers of first occurrences in areas and times from which we have many samples from. Looking at the ratio of first to all occurrences does not quite solve the problem of exaggerated number of first occurrences. Exaggeration would be the highest if we proceed from a very poorly sampled time bin to a very intensively sampled time bin. Fossil data will never be complete enough for this not to happen at all, but the uneven sample sizes at different localities may cause us to miss even more new species than on average. Sample sizes may differ from locality to locality not only due to uneven preservation or recovery, but also due to different productivities of environments. Less productive environments would generally accommodate fewer species. In addition, the number of occurrences may differ due to differences in time averaging, that is, some localities might have accumulated organismal remains for longer time than others.

Existing statistical methods for correcting for unequal sampling, including capture–mark–recapture [45–49] do not take into consideration potential variations in diversity due to varying productivity of environments. In other words, they require productivity of the environment (and thus organismal diversity) to be constant over time and attribute all differences in diversity to uneven sampling. This does not suit our purpose, where we aim to analyse species factories in the context of environmental change.

Here we make a pilot attempt to circumvent this challenge. When determining the baseline origination threshold we aim to model transitions rather than absolute states. Our model assumes that the magnitude of environmental change trends are comparable across time units, despite absolute differences in productivity. We rely on the assumptions that the carrying capacity is similar locally geographically and that it changes in a similar way over time for localities that are close to each other geographically. Thus, for each locality of interest in time and space we fit an individual localized model, which captures changes in nominal diversity taking into account its neighbouring localities. The main assumption behind this approach is that differences in diversity of spatially nearby localities are due to sampling, but differences across time units over local mean diversity is due to changes in the carrying capacity. With these spatially explicit models we aim to predict the number of first occurrences to be observed given the sample size, no matter whether the number of occurrences is large or small due to sampling, productivity or time averaging.

We model the expectation for the number of first occurrences as a function of sampling over consecutive time bins. We model the number of events, not rates. For this, we train a logistic regression model on locally weighted data. Logistic regression is a classical statistical model that internally has a linear regression, which is topped up by a sigmoid function such that the output of the model is a probability estimate between 0 and 1. The logistic regression has two input variables representing sampling intensities ‘before’ and ‘now’ (for last occurrences these would be ‘now’ and ‘after’) and one target variable denoting whether the occurrence is a first occurrence of a species in time.

**Figure 1.**
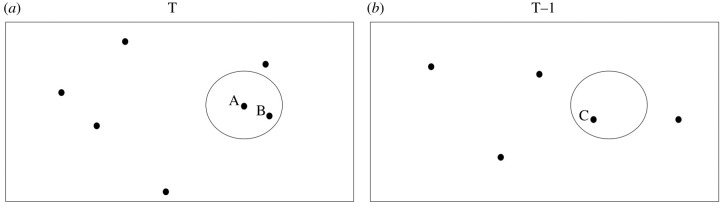
A toy example on spatial weighting: (*a*) assumed geographical area of interest at the current time unit, (*b*) the same geographical are at the previous time unit. A is the focal point (a potential species factory) for which the regression is made. Dots indicate nearby localities with fossil occurrences. A circle of a fixed radius (e.g. 500 km) is drawn around the focal locality A in figure (*a*) with another circle drawn in figure (*b*) at the same place geographically.

We weight occurrences spatially such that in the regression modeling occurrences that are close to our focal point are weighted more than occurrences that are far away. Figure 1 illustrates the spatial treatment. For this toy example, let us assume that locality A has four occurrences with one first occurrence, locality B has three occurrences and locality C (in time unit T − 1) has five occurrences. Distance from locality A to B is 400 km and C is 350 km away from the centre of the circle (locality A). Then, there are 4 + (1 − 400/500) · 3 = 4.6 weighted occurrences at time unit T and (1 − 350/500) · 5 = 1.5 weighted occurrences at time unit T − 1. We add one row (data point) for each occurrence in locality A to the overall regression model. In this case, we add four identical rows of [1.5, 4.6] as inputs and we add the vector [1, 0, 0, 0] to the overall target vector. Similar procedure is repeated for every locality in every time unit starting from MN2 so that every occurrence is included once in the dataset on which the model is fit.

The logistic regression gives us a way to estimate the probability that a given occurrence (with given sampling densities) is a first occurrence and then, given how many weighted occurrences a locality has, we can estimate how many weighted first occurrences to expect at any given grid point. Then, we estimate the probability of observing as many or more weighted first occurrences as counted at the focal grid point using the binomial distribution. Further details on modelling data and methodological choices are described in electronic supplementary material, the appendix.

### (b) Generalization to exceptional extinction patterns and species sorting

Similar principles as we described for identifying exceptional origination hotspots can be applied to analyse exceptional patterns of extinction and sorting. For extinctions, instead of first occurrences one should estimate the probability of observing as many or more last occurrences. Otherwise, the process is analogous to origination, but when creating the regression dataset, we look at occurrences in the current and next time unit (instead of current and previous), and we use occurrences within time units MN1–MQ18.

Detecting exceptional species sorting patterns (immigration and local extinction) requires an additional data processing step. We look at a geographical area around the focal point with a given radius in two consecutive time units (‘current’ and ‘previous’ for immigration, and ‘current’ and ‘subsequent’ for local extinction). For both time units, we list all the unique species observed within the focal area. Then, we remove all species from those two lists that either are first seen in the second time unit or last seen in the first time unit, i.e. we only keep species that actually exist somewhere in the world in both time units. Comparing these two lists now give us the lower bound on which species were present in the focal area in both time units and which may have become locally extinct or immigrated into it.

When creating the regression dataset to evaluate local extinction, we add a row for each species that is listed in the first time unit and include the sum of weighted occurrences in the first time unit and the second time unit as inputs of the model, and a one or a zero as the target, depending whether the species went locally extinct or not. Here, local extinction refers to species that were present in the focal area in the first time unit but not in the second, while they were observed somewhere else in the world at that time bin. The data for the immigration are prepared analogously. Similarly to origination and extinction, we fit a logistic regression with two input variables, weighted occurrences in the focal area in the two consecutive time units, and an intercept to the data.

### (c) Estimation of environmental contexts

Dental characteristics of plant eating mammal communities can be used to infer palaeoenvironmental conditions [25]. Here, we use the regression model presented by Liu *et al.* [27] that uses dental crown height (hypsodonty, HYP) and the count of longitudinal cutting edges (lophs, LOP) of molar teeth within large plant eating mammal communities as inputs to estimate the primary productivity (NPP) of the environment. As any predictions about the past, these estimates are coarse and come with uncertainties.

We weigh observations spatially the same way as described in figure 1 in attaining average distance weighted HYP and LOP values for every grid point (where HYP and LOP scores are available in NOW database). HYP values are ordinal, roughly corresponding to the ratio scale. LOP values are counts. Taking the average over HYP and LOP is meaningful biologically and computationally.

### (d) Implementation

The data and analysis pipeline is available on GitHub (https://github.com/jaksticks/NOW_codes). The code is in Python. We used the statsmodels package for Python to perform all the regression analyses. The analysis is scripted in Jupyter notebooks.

For our main analysis, we used a radius of 500 km for the spatial aggregation. We also performed the same analyses using a 100 km radius, the corresponding complementary plots are available in the code repository.

## 4. Results

### (a) Palaeoenvironments

**Figure 2.**
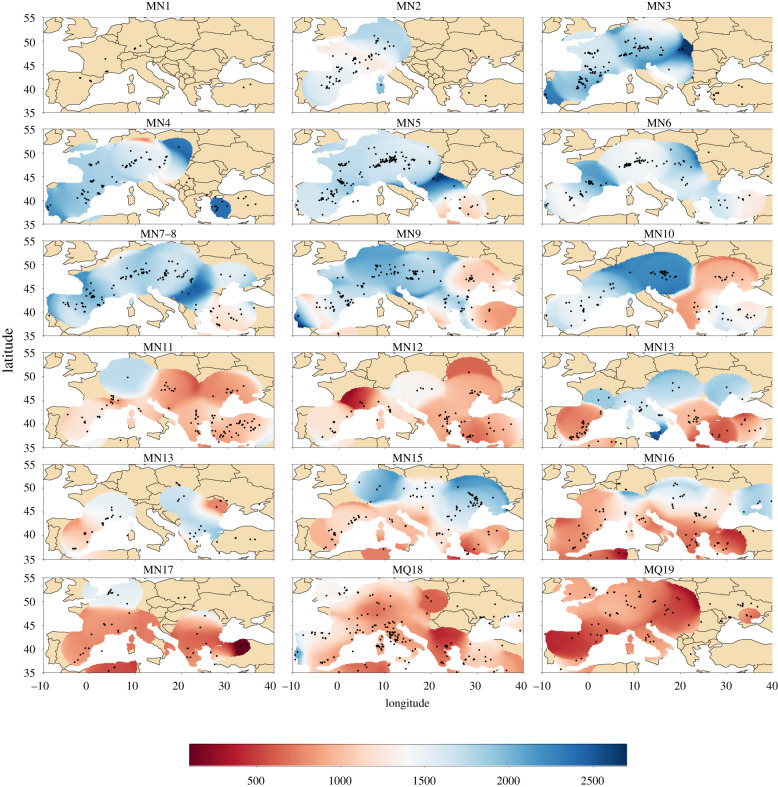
Net primary productivity (with units g Cm^−2^ yr^−1^) estimated from dental traits of large plan eating mammal community data. Blue indicates areas with high estimated productivity and red indicates areas with low productivity. All spatial grid points with at least two weighted occurrence is displayed. Black dots indicate the location of all fossil localities designated for the given time unit.

Figure 2 maps estimates of environmental productivity. We observe that Early Miocene eastern Europe (Turkey in particular) begins to drop in NPP before the rest of Europe. This becomes particularly evident in MN9 and MN10. By MN11 and MN12 most of Europe has also dropped considerably in NPP. From MN13 to MN16 there appears to be a fairly clear division between more lush environments (higher estimated NPP) in central or northern central Europe, whereas western and eastern Mediterranean remain arid (low in estimated NPP). From MN17 onwards we observe further overall aridification.

**Figure 3.**
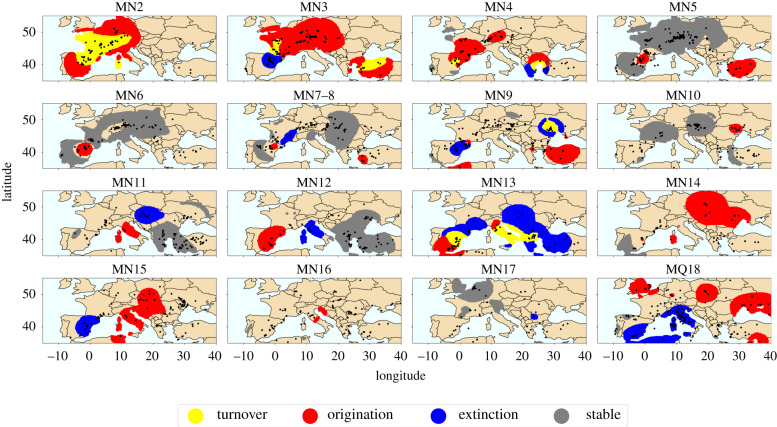
Diversity patterns. Red indicates areas with statistically significant (*p* < 0.05) number of weighted first occurrences (origination). Blue indicates areas with statistically significant (*p* < 0.05) number of weighted last occurrences (extinction). Yellow indicates areas with statistically significant (*p* < 0.05) number of weighted first and last occurrences (turnover). Grey indicates areas with statistically small (*p* > 0.95) number of weighted first and last occurrences (stable). All spatial grid points with at least one weighted occurrence is displayed. Black dots indicate the location of all fossil localities designated for the given time unit.

**Figure 4.**
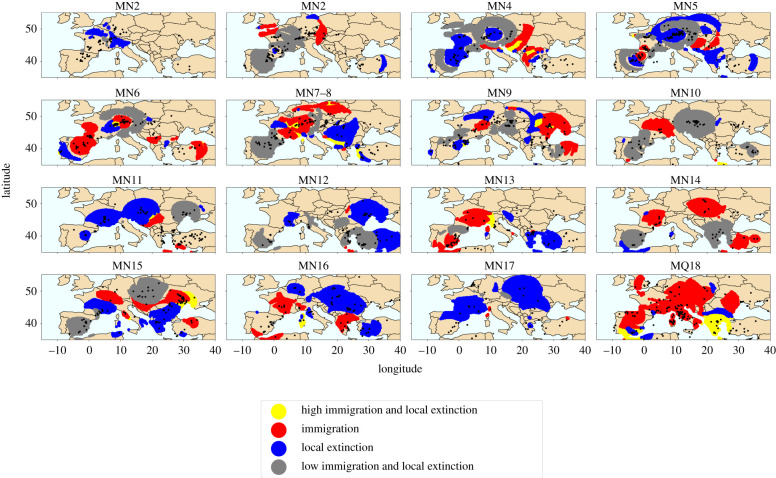
Sorting patterns (local appearance and local extinction). Red indicates areas with statistically significant (*p* < 0.05) immigration. Blue indicates areas with statistically significant (*p* < 0.05) local extinction. Yellow indicates areas with statistically significant (*p* < 0.05) immigration and local extinction together. Grey indicates areas with statistically small (*p* > 0.95) immigration and local extinction together. All spatial grid points with at least one weighted occurrence in the focal, preceding and subsequent time unit is displayed. Black dots indicate the location of all fossil localities designated for the given time unit.

Figures 3 and 4 depict identified origination, extinction and sorting hotspots. Figures of origination, extinction, immigration and local extinction separately are given in electronic supplementary material.

**Figure 5.**
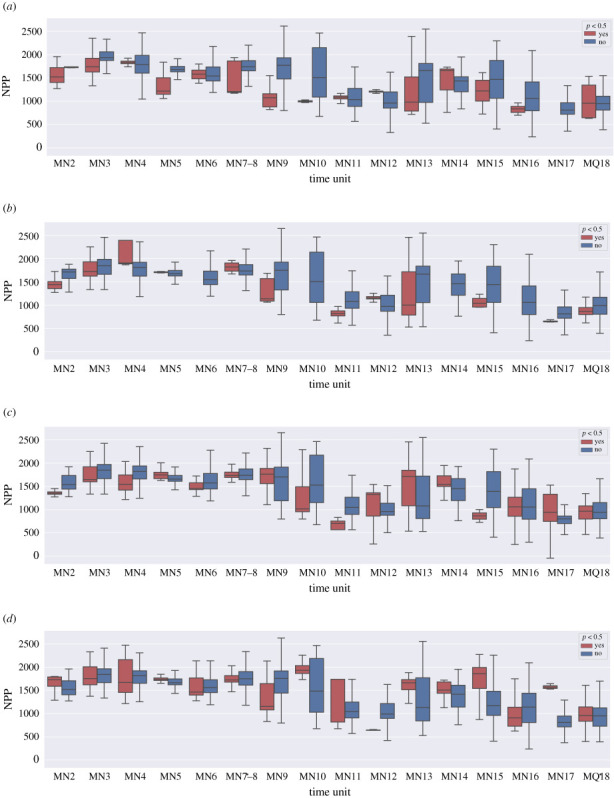
Comparison of origination, extinction and species sorting hotspots versus non-hotspots with respect to net primary productivity (NPP). In each plot, two boxplots are shown for each time unit: red for a given hotspot type and blue for corresponding non-hotspots. The calculations include all spatial grid points, which have at least two weighted occurrences with the relevant HYP and LOP data available. (*a*) Speciation. (*b*) Extinction. (*c*) Local extinction. (*d*) Immigration.

In the earlier part of the Miocene, up to and including MN10, origination hotspots are primarily on the more arid side of the environmental gradient (figure 5*a*). Extinction hotspots appear quite dynamic throughout (figure 5*b*). Meanwhile, the estimated productivity for sorting hotspots is mostly in par with the mean for non-hotspots up to and including MN7–8 (figure 5*c*,*d*). Immigration hotspots show quite wild environmental fluctuations between MN9 and MN12, and then up to MN17 they tend to be found on the more lush side compared to non-hotspot areas. Local extinction also shows much more variation (compared to non-hotspots) after and including MN10 than it did prior to that time bin.

### (b) Faunal dynamics in the Early Miocene

The Early Miocene (MN2–MN4) displays widespread origination hotspots occurring (figure 3). While this may be partially due to modest representation of older records in Europe in the NOW data, the identified hotspots in these Early Miocene times extend far beyond the region with increasingly dense sampling (electronic supplementary material, figure S12). In particular, central Europe as well as Turkey and Greece continuously appear as hotspots, most prominently in MN3. At the same time bin (MN 3) central Europe appears as an immigration hotspot (figure 4). The map of productivity estimates suggests that during MN3 central Europe might have had slightly more productive environment than the surrounding areas and also more productive than in the previous time step. Turkey and Greece lack large mammal data to display environmental estimates for MN3, but the territory appears much more productive than the European context in MN4 and also partially lights up as an origination hotspot.

The red area in MN2 in the Iberian peninsula does not have central localities (no black dots), but lights up red due to neighbouring localities within 250 km (500 km radius) showing exceptionally many new species. Perhaps in future work this could be remedied with an exponentially decaying weight scheme for the occurrences or by cutting out grid points that are too far away from any localities.

Figure 5 compares environmental estimates at identified hotspots against regular areas over time. We see that overall statistically in the Early Miocene origination hotspots tend to occur at the more arid end of the environment, thus the visual pattern in central Europe and Turkey makes an exception. An interesting observation emerges when comparing trends, we can see that the estimated productivity around origination and extinction hotspots steadily increases over MN2–MN4 against more or less steady level of productivity for regular sites at the same time period. At the same time, immigration patterns show the reverse—immigration hotspots stay at the steady level while regular spots show an upward trend. Intuitively, this makes sense, origination and extinction hotspots seem to be at the frontier of environmental change, whereas immigration hotspots are those places that carry on the previous conditions while globally the environment changes; in other words, refugia.

### (c) Faunal dynamics in the Middle Miocene

The Middle Miocene appears as time of stability in terms of origination and extinction. One prominent area is northeast Spain, which appears as an origination hotspot already since MN4 and carries onwards as a hotspot all the way through MN7–8. This might be related to a known diachrony of the MN system, with the temporal time correlates of MN units 4–6 being consistently younger in Spain than in the eastern Mediterranean or central Europe, so that some actually later-occurring taxa might appear to occur ahead of their time in Spain [50]. Despite this offset, Turkey also displays recurring origination hotspots in the same time frame, most prominently in MN5. Thus it would clearly be premature to attribute these patterns to diachrony alone.

While the majority of Europe remains relatively stable in terms of origination, many more hotspots of sorting appear, which are also dynamically intermixed during this period. Migration patterns that start in MN4 continue through MN5—geographically central Europe acts as a source area, while southeast and (later) southwest act as sink hotspots. It seems this could be a natural succession of the Early Miocene species factory in central Europe; the ‘manufactured’ species now move away. Environmentally, we indeed see (figure 2) that the directions of dispersal preserve slightly higher productivity in MN5, especially the southwest.

In MN6 and even more so in MN7–8 the directions of species sorting hotspots reverse—the central north becomes the immigration hotspot. However, there is no clear environmental contrast in association with this. The box plots comparing environmental conditions of sorting hotspots with non-hotspots are more dynamic than in the Early Miocene, the conditions progress to more arid in MN6, but then go back to the Early Miocene level in MN7–8.

Overall, distribution of conditions of sorting hotspots against background conditions strongly suggest that species sorting tracks stable conditions. It does not mean that conditions need to remain stable at the same place.

We rather see that when many sorting hotspots occur (like in the Middle Miocene here), those hotspots, especially the immigration hotspots, conserve the environmental conditions of previous times, acting as refugia [51].

### (d) Faunal dynamics in the Late Miocene

The known Vallesian pattern is seen in MN9 western Europe, but this appears to be a local, rather than a continent-wide event, which is in line with recent research [52]. Eastern Europe, however, does exhibit another extinction hotspot in MN9 (figure 3). Both Spain and eastern Europe spots show as notably more arid than the rest during the time period (figure 2). At the same time Turkey, which also shows as extremely arid in MN9, appears as an origination hotspot. After several time units of stability, associated with higher productivity estimates during MN10–MN12, in MN13 Turkey displays large local extinction and permanent extinction hotspots, which might be interpreted as biodiversity sinks. The estimates show that in MN13 Turkey is extremely arid along with Spain, while the northern territories across Europe return to more productive environments.

**Figure 6.**
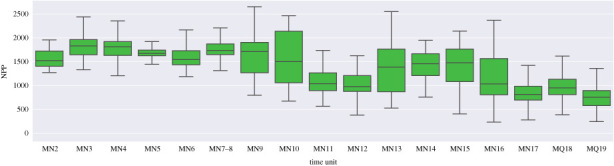
Net primary productivity over time estimated from dental traits of large plant eating mammal communities. The calculations include all spatial grid points, which have at least two weighted occurrences with the relevant HYP and LOP data available. (Online version in colour.)

Overall, the Late Miocene has the most a notable dip in estimated aridification in MN11 and MN12 (figure 6). In MN11, both local and permanent extinction hotspots are by a wide margin environmentally harsher than the rest of the localities (figure 5). Those might be interpreted as sink areas. Those highly prominent local and permanent extinction hotspots in MN11 cover large areas in central-southern Europe (figures 4 and 3). Italy at the same time acts as an origination hotspot, quickly becoming an extinction hotspot in the following MN12 time unit. This probably reflects the presence of insular, highly endemic faunas in Italy at this time.

The origination hotspot trend in the Late Miocene is similar to the pattern observed in the Early Miocene. Origination hotspots start the most arid as compared to the background conditions and steadily increase in productivity while the background conditions follow the European v-shaped trend in average aridity. In other words, it seems that the defining feature of species factories to be exceptional environmentally and represent conditions that soon will become wide spread broadly holds. In the Late Miocene, species factories start arid and then become like the rest. It is more difficult to make clear conclusions from immigration contrasts at the same period, but at least in MN10, which is the first notable time step towards more arid conditions, origination hotspots are notably more arid than the background, while immigration hotspots are notably less arid than the background (figure 5).

### (e) Faunal dynamics in the Pliocene and Pleistocene

The final stage of our analysis firstly (MN14–MN16) display highly polarized environments, continuing the pattern from MN13—productive in the north and more arid in the south, and then at the latest time stages (MN17–MQ18) everything becomes quite arid. In MN14, the productive northeast acts as a large origination hotspot, to an extent continuing into MN15 and shifting south in MN16 (figure 3). Again, MN16 is just before everything gets more arid and at that unit origination hotspots are in harsher conditions than the rest of the localities (figure 5). At the same time of polarized Europe MN14 and MN15 show notable immigration hotspots in central Europe (figure 4) that appear notably more productive in terms of NPP than the background conditions (figure 5). In MN16, when the last margins of the productive north begin to disappear central Europe and especially central eastern Europe become one huge local extinction hotspot (figure 4). While the productivity maps look like the Black Sea shores in MN16 might have acted as a refugium, the question is why species so massively go locally extinct and why is this not becoming a permanent extinction hotspot neither in MN16 nor in MN 17 (figure 3) when the last green colour disappears from NPP estimates (figure 2).

The final periods of the analysis are as quiet as they are dramatic. MN17 is extremely stable in terms of origination and extinction, but shows massive local extinction hotspots. An open question is in what other places they continuing after local extinctions. MQ18 shows extinction hotspots in the south and several large origination hotspots in the north, as well as massive immigration hotspots. Could those that left in MN17 now be getting back?

### (f) Species longevity and occupied area

Considering only species that had a first and a last occurrence within MN2–MQ18 in the study area, we find that the average age of origination hotspot species (species that had at least one first occurrence in an origination hotspot) is 3.5 Myr, whereas non-hotspot species (species without any first occurrence in an origination hotspot) only had an average observed lifespan of 2.5 Myr. If we for a moment disregard species that occurred in a single time unit, then the corresponding average longevity of the remaining species would be 5.0 and 4.1 Myr, respectively.

We further estimated the geographical ranges of each species over their lifetime. Specifically, for each species and time unit, if a given species had three or more occurrence locations, we calculated the area of the convex hull of those locations. When only two locations existed for a given species and time unit, we calculated the distance between those locations and assumed a 10 km wide rectangle between those two locations for which we calculated the corresponding area. If there was only one location for a given species and time unit, we assumed the species range to be zero for that instance. We found that origination hotspot species occupied an average area of 135 000 km^2^ and non-hotspot species occupied an average area of 86 000 km^2^ over their longevity. Excluding single time unit species, we found that hotspot and non-hotspot species’ average occupied areas were 229 000 km^2^ and 177 000 km^2^, respectively.

We also looked at average longevity and occupied areas for species that had a last occurrence in an extinction hotspot. We found that the mean longevity was 3.1 and 2.9 Myr for hotspot and non-hotspot species, respectively. Removing single time unit species, we found that the mean longevity for hotspot species was 4.6 Myr and for non-hotspot species it was 4.5 Myr. The mean occupied area for hotspot species was 176 000 km^2^ and for non-hotspot species it was 68 000 km^2^. Removing single time unit species, we found that the mean occupied area for hotspot species was 333 000 km^2^ and for non-hotspot species it was 125 000 km^2^.

## 5. Discussion

Species factory as an evolutionary concept has existed as part of the informal discourse of evolutionary palaeontologists for a long time [7,53], but apart from our earlier study in adaptive dynamics [54] appears not to have been defined explicitly or formalized computationally. The term has been used in a broad sense for the source area of exceptionally many species [6]. Empirically the concept has been applied in several contexts to explain the dynamics of mammalian faunas—the latest Middle Miocene origins of the Pikermian palaeobiome in the Sub-Parathetyan province [7], the Pliocene origin of Pleistocene megafauna in the Tibetan Plateau [9] and the rise of arid-adapted fauna in the Plio-Pleistocene of the eastern African Turkana Basin [8]. How prevalent the phenomenon is across the world and what macroevolutionary mechanisms may be behind it has remained unclear.

Our attempt to define and account for species factories and related concepts in a systematical way at a large spatial and temporal scale shows that the phenomenon can be detected computationally. The phenomenon, of course depending on the thresholds for deciding what is exceptional, appears to be more common than the few anecdotal cases known previously. Based on the patterns resulting from our analysis, it seems that species factories are by and large a matter of being present in locations that represent conditions that are about to become widespread. Whether adaptations leading to these originations are somehow special or simply a matter of lucky placement remains an open question but from our pilot analysis we see that species from recognized species factories do tend to live longer and become more widespread than species on average. We hope follow up studies will address this question in more detail.

A refugium is the macroevolutionary counterpart of species factory. The term refers a location that supports relict populations of previously more widespread species. The concept has been commonly used to describe survival through and recovery from adverse conditions, such as glacial–interglacial cycles. Here we label as refugia areas that receive abnormally many survivors that have become locally extinct elsewhere. Such areas can be a long or short term refugia depending on what happens next. The absence of extinction hotspots along with refugia implies that a refugium was successful; the species did not come there to die, but to survive through bad times and carry on.

From this perspective, species sorting appears as a more complicated phenomenon than origination, not expected to follow any simple pattern. It draws on availability of immigrants, availability of dispersal routes, the absence of competitors and other such factors that are challenging to account for analytically.

The interpretation of the extinction hotspots is less clear, but from the perspective of species survival they could be either well-sampled places of bad luck at bad times, or they could be short-lived refugia. An open question concerns the large differences in species range between extinction hotspot and non-hotspot species. Why do common species appear there last before extinction, while rare species do not? Could this be a matter of broad versus local adaptation?

Another open question is what proportion of species originate from ‘species factories’. While we showed that species factories exist, we could not within the methodology of our pilot analysis assess the prevalence of the factories. How common are they? Clearly, species factories cannot be as common a mechanism as the majority rule postulated by Vermeij & Dietl [2]. A species factory is an exception but not an unexpected one. It does require a certain configuration of environmental changes over time and space but over millions of years of environmental change that particular configuration will inevitably occur from time to time.

The computational methods put together in this paper allow us to find exceptional patterns from fossil occurrence data. To the extent of the coverage that the fossil record can give us, we expect these patterns to be robust with a natural level of noise present in the data. The tailored computational solution involved many design choices. As always, choices might have been different, the solution that we report here was driven by feasibility and transparency of the approach. We did not explicitly model sampling effects on diversity versus true variation in diversity due to changing carrying capacity. This is an open question for which we do not have resolutions to offer yet. Nonetheless, we hope that we captured both effects implicitly to a crude approximation via locally weighted models. Rather than modelling sampling versus carrying capacity explicitly, the idea of the current study was to model how many events are expected assuming that the carrying capacity was similar locally geographically and did not change fast in time. In this case, we expected the localized regression approaches with local weighing to take care of potential unevenness in sampling.

The hotspots that we found open broader research directions for future: what is the community composition, what are the environmental conditions, are there common features between different hotspots? Do species from local extinction hotspots tend to move to immigration hotspots? Identifying and studying these different hotspot areas can help us learn more about important evolutionary and ecological mechanisms over geological time scales.

## 6. Conclusion

We set out to identify species factories computationally. For that we developed a regionally informed statistical approach to detect exceptional places and times of species origination, extinction and sorting in the fossil record. Applying this approach to the mammalian fossil record of the Late Cenozoic we found that early hotspots were characterized by species adapted to environments that subsequently became more common, giving these hotspot species a potential selective advantage. Our pilot results also suggest that species originating in hotspots had on average longer longevity and larger geographic ranges than other species.

Species factories thus appear to be a real phenomenon. Our analysis suggests that species factories can be found particularly in synch with gradual but persistent directional changes of environmental conditions. A species from the factory is thus a lucky species—appearing at the right time in the right place, and with the right set of evolutionary raw material for the new adaptations required.

## Data Availability

All scripts used in this study are openly accessible through https://github.com/StochasticBiology/boolean-efflux.git. The data are provided in electronic supplementary material [55].
